# Tuning layered superstructures in precision polymers

**DOI:** 10.1038/s41598-020-68927-x

**Published:** 2020-07-21

**Authors:** Varun Danke, Sophie Reimann, Wolfgang H. Binder, Gaurav Gupta, Mario Beiner

**Affiliations:** 1 Fraunhofer Institute for Microstructure of Materials and Systems IMWS, Walter-Hülse-Straße 1, 06120 Halle (Saale), Germany; 2 Chair of Macromolecular Chemistry, Faculty of Natural Sciences II, Institute of Chemistry, Martin-Luther-University Halle-Wittenberg, 06120 Halle (Saale), Germany; 3 Faculty of Natural Sciences II, Martin-Luther-University Halle-Wittenberg, Heinrich-Damerow-Straße 4, 06120 Halle (Saale), Germany

**Keywords:** Nanoscale materials, Soft materials, Structural materials, Polymer chemistry

## Abstract

An approach to influence and control layered superstructures by varying the methylene sequence length between two consecutive functional groups in linear precision polymers containing 2,6-diaminopyridine (DAP) groups is presented. Layered superstructures with repeating units involving three monomeric units along the chain direction with very high coherence lengths upto 110 nm are observed in case of shorter alkyl segments, (16 and 18 ${\hbox {CH}}_{2}$ units), while more conventional layer superstructures incorporating only one monomer are found for related polymers with 20 ${\hbox {CH}}_{2}$ units per methylene sequence. A building block model explaining the unusually large periodicity of three monomeric units is proposed wherein layers containing crystalline or amorphous methylene sequences occur in different combinations. Occurrence of different layered structures depending on crystallization conditions, methylene sequence length as well as functional group type is explained by a competition of H-interactions between the DAP groups and the van der Waal forces between the hydrophobic methylene groups.

## Introduction

Precise sequence control over chain constitution and architecture to obtain well-defined, tailor-made morphologies has always been one of the grand challenges in polymer synthesis. It has almost been a century since polyethylene was invented by accident in 1933 and over 50 years since organo-metallic Ziegler-Natta and Phillips catalysts first paved the way for efficient control over branching in polyethylene. Recently, use of advanced techniques to program different functional groups at well defined locations with efforts to replicate nature’s precise synthesis and create multifunctional systems has gained momentum^1–3^. Advances in polymer synthesis in the form of acyclic diene metathesis (ADMET) polymerization^4^ have allowed for remarkable control over chain constitution in terms of precise placement of functional groups along a linear polyethylene chain. The incorporation of such functional groups at well defined locations along the backbone could introduce interesting properties due to the chemical nature and interactions between the groups^5^. A number of functional groups such as alkyl branches^6^, 2,6 diaminopyridine (DAP)^7^, boronic acid and ester^8^, phosphoester^9^, phosphonic acid^10^, azobenzenes^11^, or halogens^12^, can be placed along the polyethylene chain. The freedom to elegantly engineer molecules with accurately controlled sequences has opened up an untapped reservoir of application relevant materials. Biomimetic approaches towards engineering in-vivo biocompatible materials incorporating various other regularly spaced groups along a flexible aliphatic chain have received considerable interest^13,14^. According to a recent study, precision polyethylenes containing regularly spaced acidic groups can be used for efficient ion transport^5^. Similarly, well-ordered arrangement of ring-like pendant groups such as perylene bisimide or terfluorene groups could be used to design advanced organic opto-electronic devices^15,16^.

Property optimization often demands efficient control over morphology which requires an in-depth understanding and control of the underlying structure formation mechanisms. Most of the investigated precision polymers are known to exhibit layered states on the nano-scale on account of alternating arrangement of the methylene sequences and the functional groups. The packing of the methylene sub-units within the layers depends upon the size and nature of the adjacent functional group as well as on the length of the methylene sequence. For example, bulky ring-like groups lead to liquid-like morphologies at ambient temperatures wherein the methylene sequences remain amorphous^17,18^. Conversely, smaller pendant atoms such as halogens cause minor distortions in the native crystal-like packing of the methylene sequences^12^. It is well known that inter-molecular interactions play a vital role in the hierarchical structures in complex natural systems like proteins as well as other systems of industrial importance such as polyamides where intermolecular hydrogen bonds between neighboring amide groups were found to dictate overall crystallization^19^. This raises fundamental questions regarding the competition between the packing tendencies of the individual sub-units viz. functional groups and methylene sequences for structure formation processes in complex systems^20,21^.

**Scheme 1:**
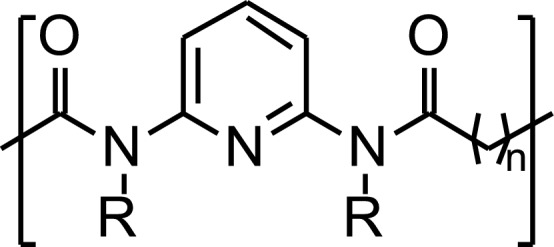
Monomeric repeating unit of the investigated UDAPS series. n=16, 18 or 20.

In this study, we demonstrate the importance of supramolecular interactions in governing the structural features of precision polymers incorporating regularly spaced functional groups in linear polyethylene. It is demonstrated that the used 2,6-diaminopyridine (DAP) group capable of introducing interactions via H-bonding^22^ or pi-stacking^23^ can stabilize different layered superstructures containing up to three monomeric repeating units (refer Scheme 1) along the chain axis. Varying the DAP to methylene units ratio (methylene spacer length) allows for tuning the nature of the layered structures. A competition of the energetic contributions of the hydrophobic and hydrophilic interactions between the sub-units controls the structure formation.

## Results and discussion

Structure formation in a series of precision polymers having a 2,6 diaminopyridine group (DAP) group placed regularly after every 16th, 18th or 20th methylene unit (UDAPS16, UDAPS18 and UDAPS20 respectively) as shown in Scheme 1 is studied using temperature dependent intermediate angle X-ray diffraction (IAXD) during step-wise cooling from the melt state. The polymers were synthesized using acyclic diene metathesis polymerization (cf. section “Materials and methods”).

**Figure 1:**
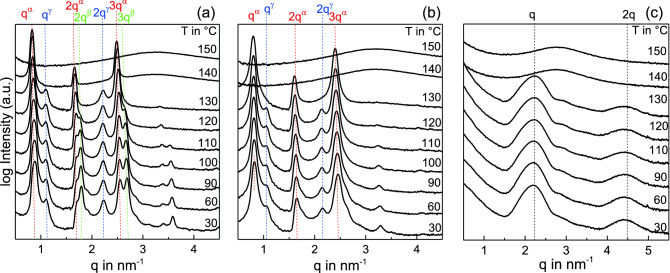
Intermediate angle X-ray diffraction (IAXD) patterns for (**a**) UDAPS16, (**b**) UDAPS18 and (**c**) UDAPS20 during stepwise cooling from the melt. The curves are vertically shifted for the purpose of visibility.

The structure formation process in UDAPS16 commences at $140^{\,\circ } \hbox {C}$ where three sharp peaks are observed (Fig. 1a). The first peak seen at $\textit{q} \approx 0.833 \, {\hbox {nm}}^{-1}$ labeled as $q^{\alpha }$ together with the presence of its higher orders ${\textit{2q}}^{\alpha }$ and ${\textit{3q}}^{\alpha }$ indicate a layered morphology with an interplanar spacing of $d=2 \pi /q^{\alpha } \approx 7.57 \, \hbox {nm}$. Upon further cooling to $120^{\,\circ } \hbox {C}$, two additional sharp reflections develop with peak positions at $q \approx 1.1 \, {\hbox {nm}}^{-1}$ and $\textit{q} \approx 2.2 \, {\hbox {nm}}^{-1}$ labeled as $q^{\gamma }$ and ${\textit{2q}}^{\gamma }$ respectively. They correspond to another layered phase having an interplanar spacing of $d^{\gamma } \approx 5.7 \, \hbox {nm}$. Below $110^{\,\circ } \hbox {C}$, another layered phase can be observed with a spacing of $d_{\beta } \approx 7 \, \hbox {nm}$. Second and third order peaks at ${\textit{2q}}^{\beta }$ and ${\textit{3q}}^{\beta }$ are seen in the IAXD pattern while the first order peak at $q^{\beta } \approx 0.9 \, {\hbox {nm}}^{-1}$ is only resolved in a SAXD pattern (Fig. 3a inset). Surprisingly, the interplanar spacing of both $\alpha$ and $\beta$ forms indicates that the periodicity of this layered structure corresponds approximately to three monomeric repeating units ($l_{m} \approx 2.63 \, \hbox {nm}$ for UDAPS16^18^). Similar behavior with two different superstructures incorporating three monomeric repeating units is found in UDAPS18 (see Fig. 1b). The $\alpha$ form with layer spacing of 7.95 nm is formed initially during cooling at $130^{\,\circ } \hbox {C}$ followed by the $\gamma$ form at $120^{\, \circ } \hbox {C}$ showing a layer spacing of 5.87 nm. However, unlike the case in UDAPS16, a prominent growth of the $\beta$ form is not observed.

Interestingly, the situation in UDAPS20 is drastically different as compared to UDAPS16 and UDAPS18 (Fig. 1c). The observed layer spacings indicate the absence of superstructures in this slightly modified precision polymer. Upon cooling below $130^{\,\circ } \hbox {C}$, a broad reflection evolves, with the peak maximum at $q \approx 2.2 \, {\hbox {nm}}^{-1}$ corresponding to a spacing of 2.85 nm, which is close to the length of one monomeric repeating unit. The presence of the second order reflection indicates a layered structure. Obviously, the addition of only two more methylene groups results in the disappearance of the superstructures incorporating more than one repeating unit. At this point it can be hypothesized that the higher concentration of supramolecular interactions introduced by the DAP groups in UDAPS16 and UDAPS18 as compared to UDAPS20, which has slightly higher aliphatic content, are responsible for this superstructure.

**Figure 2:**
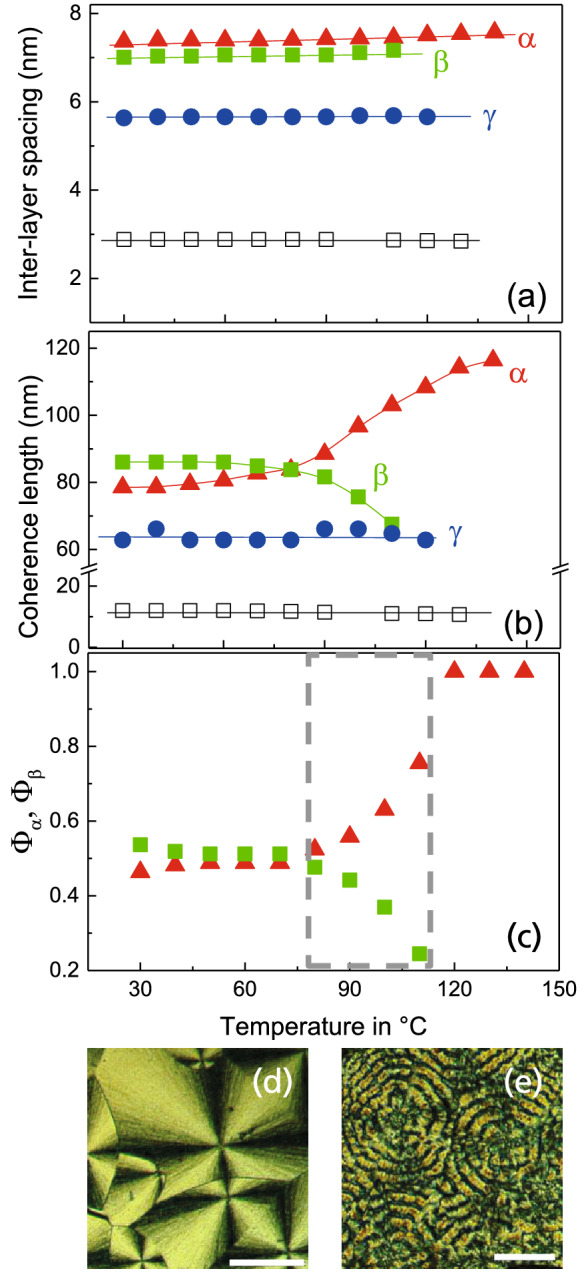
Temperature dependent (**a**) layer spacings and (**b**) coherence lengths ($L_{\textit{coh}}$) of the $\alpha$ (red triangles), $\beta$ (green squares) and $\gamma$ (blue circles) forms in UDAPS 16 and UDAPS20 (open squares). Note that for reasons related to peak resolution, the third order layer reflections ${\textit{3q}}^{\alpha }$ and ${\textit{3q}}^{\beta }$ of ${\alpha }$ and ${\beta }$ form respectively and second order layer reflection ${\textit{2q}}^{\gamma }$ of the ${\gamma }$ form were used for the evaluation. (**c**) shows the temperature dependent intensity fractions $\phi _{\alpha }$ and $\phi _{\beta }$ calculated from the *3q* reflection for $\alpha$ (red triangles) and $\beta$ (green squares) forms during cooling. (**d**,**e**) show the polarized optical micrographs for UDAPS16 and UDAPS20 respectively. The scale bar for (**d**,**e**) is 200 µm and 40 µm respectively.

In this paragraph important structural differences between UDAPS16 and UDAPS20 as representative examples will be highlighted. The temperature dependence of the Bragg spacings d in UDAPS16 and in UDAPS20 measured during cooling are shown in Fig. 2a. The calculated spacings confirm that the repeating unit of layered structures in the $\alpha$ and $\beta$ forms of UDAPS16 include three monomers along the layer surface normal. Whereas, the $\gamma$ form has a layer spacing which seems to be in-between the extended lengths of two and three monomeric repeating units. In contrast, UDAPS20 has a layer spacing corresponding to one monomeric sequence length in the entire temperature range. Additional insights emerge on comparing the temperature-dependent coherence lengths as taken from Scherrer’s equation in the form $L_{\textit{coh}} = 2 \pi /\delta q$ with $\delta$q being the full width at half maximum (see Fig. 2b). $L_{\textit{coh}}$ quantifies the number of repeating units in a crystal along a certain direction. All the layered forms in UDAPS16 exhibit extremely large coherence lengths, the largest by the $\alpha$ form. In the temperature window $120^{\,\circ } {\hbox {C-90}}^{\,\circ } \hbox {C}$ the drop in coherence length of $\alpha$ form from $L^{\alpha }_{\textit{coh}} \approx 115 \, \hbox {nm}$ to $L^{\alpha }_{\textit{coh}} \approx 80 \, \hbox {nm}$ is complemented by an increase in that of the $\beta$ form from $L^{B}_{\textit{coh}} \approx 68 \, \hbox {nm}$ to $L^{B}_{c} \approx 85 \, \hbox {nm}$. This indicates that there is most likely a $\alpha$-$\beta$ solid–solid transition occurring in this temperature window. This is supported by the temperature dependence of the integrated intensities ($\phi _{\alpha } = I(3q^{\alpha })/I(3q^{\alpha })+I(3q^{\beta }$) and $\phi _{\beta } = I(3q^{\beta })/I(3q^{\alpha })+I(3q^{\beta })$) of the the third order layer reflections ${\textit{3q}}^{\alpha }$ and ${\textit{3q}}^{\beta }$ shown in Fig. 2c. During cooling, the decrease in $\phi _{\alpha }$ within the temperature window $120^{\,\circ }\hbox {C}$–$90^{\,\circ }\hbox {C}$ is complemented by a simultaneous increase in $\phi _{\beta }$ as expected for an $\alpha$-$\beta$ solid-solid transition. This phase transition is also seen in the same temperature window during subsequent heating (c.f. Supplementary Information) indicating that the process is reversible. The coherence length of the $\gamma$ form is also high ($\approx 62 \, \hbox {nm}$) and remains fairly constant over the entire temperature range. It is also noteworthy to mention that the superstructures of UDAPS18 also show extremely large coherence lengths similar to UDAPS16 which is also apparent from the narrow peak widths of the Bragg reflections. In contrast, UDAPS20 is characterized not only by the absence of superstructures, but also by a significantly shorter and constant coherence length of $L_{{\textit{coh}}} \approx 12 \, \hbox {nm}$ over the entire temperature range.

**Figure 3:**
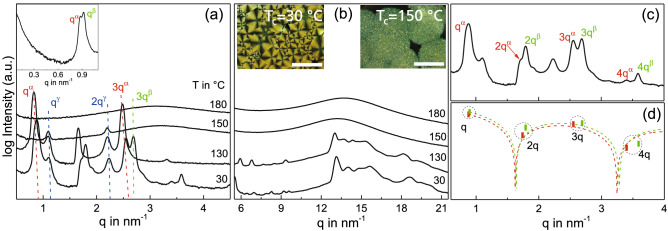
(**a**) IAXD patterns at selected temperatures during heating showing the presence of three layered states in UDAPS16. The inset shows a measurement performed in the lower *q* range which resolved the first order reflections of the $\alpha$ and $\beta$ forms. (**b**) shows the corresponding WAXD patterns at the respective temperatures. The IAXD and WAXD curves in (**a**,**b**) are vertically shifted for the purpose of visibility. The left and right insets in (**b**) show polarized optical micrographs for samples isothermally crystallized at $30^{\,\circ } \hbox {C}$ and $150^{\, \circ } \hbox {C}$ respectively, both with a scale bar of 500 µm. (**c**) Shows the room temperature IAXD pattern at $30^{\,\circ } \hbox {C}$ for UDAPS16 after cooling from the melt with the corresponding layer reflections indexed while (**d**) shows the corresponding form factor fit of peak intensities for a lamellar morphology for $\alpha$ (red) and $\beta$ (green) form.

We interpret the extremely large coherence lengths (Fig. 2b) together with the absence of the SAXD peak (Fig. 3a inset) which typically corresponds to a ’long period’, as an indication for an unusually perfect periodicity of the layers containing a negligible fraction of the sample which is not packed within the layered structure. The absence of this reflection in the case of a precisely sulfonated polyethylene was taken as a measure of an unusually large crystal size^5^. However, this reflection is seen in UDAPS20 as well as in analogs in which the DAP-group is protected by replacing the H-atom with a ${\hbox {CH}}_{3}$ group (refer Supplementary Information). This ’protection’ of the DAP group prevents hydrogen bonding between neighboring DAP groups. Therefore, we infer that a comparatively higher concentration of the H-bonds is the most probable reason for the extremely long-range ordered structures seen in UDAPS16 and UDAPS18.

Moreover, the spherulitic structure also shows distinct differences between UDAPS16 and UDAPS20 (Fig. 2d,e). UDAPS20 shows ring-banded structures in the polarized optical micrographs which are often considered to be a product of lamellar twisting^24^ due to unbalanced stresses from the opposite ends of chain folding surfaces^25,26^.

The marked differences in the structures seen in UDAPS16, UDAPS18 and UDAPS20 are most likely a consequence of the supramolecular interactions introduced by the DAP groups. The DAP groups can build intermolecular hydrogen bridges while the pyridine ring moeity can promote $\pi$–$\pi$-stacking^27,28^. The 2,6 diamino pyridine itself is known to crystallize in the form of different polymorphs^29^. The ring N-atom together with the O-atoms may act as acceptor sites while the amino H-atoms act as donors. The H-bonding behavior of 2,6 diaminopyridine based molecules reportedly showed a three-point hydrogen bond network which was shown to recognize flavins in solutions^27^. It is therefore, also plausible that DAP units could crystallize in the form of rigid planes within the layers. It can be argued that the missing superstructure in UDAPS20 is most likely because of the higher hydrophobic energetic contributions introduced by the additional ${\hbox {CH}}_{2}$ units. Highly ordered superstructures observed in UDAPS16 and UDAPS18 might be a result of the higher density of the supramolecular interactions of the DAP groups. In general, it can be expected that longer methylene sequences or lower frequency of the DAP groups would promote crystallization of PE-like domains due to a greater contribution from the van der Waal interactions from the aliphatic segments as seen for other related systems^17,30,31^. This points towards a competition between the packing tendency of the two sub-units viz. DAP groups and methylene sequences opening up the opportunity for tuning the long range order.

Such competing effects were also reported in precision polyethylenes incorporating a regularly placed phenyl group^32^. For lower methylene sequence lengths between two consecutive phenyl rings, the crystallization behavior is governed by the $\pi$–$\pi$-interactions between the rings whereas for longer methylene sequence runs, the $\pi$–$\pi$-interactions are strongly hindered. Similar effects are also known to occur in other related systems such as comb-like polymers with rigid backbones wherein the often fully aromatic backbones have a tendency to show $\pi$–$\pi$-stacking while the alkyl side chains can crystallize within the backbone-induced constraints only when long enough^20,21^. The competition between the backbone and side chain ordering was found to have an effect on the opto-electronic properties^33^.

As the structural differences in the two extreme cases considered above and the underlying reasons become clearer, we now address the differences between the superstructures $\alpha$, $\beta$ and $\gamma$. In the IAXD pattern of UDAPS16 during heating, all the three forms-$\alpha$, $\beta$ and $\gamma$ are seen at $30^{\,\circ } \hbox {C}$ as shown in Fig. 3a. At $130^{\,\circ } \hbox {C}$, only forms $\alpha$ and $\gamma$ are observed while at $150^{\, \circ } \hbox {C}$ only $\gamma$ form is present prior to final melting. The relatively sharp peaks of the $\gamma$ form point toward a long range layered order but the absence of any reflections at higher scattering vectors in the WAXD region as seen in Fig. 3b indicates an absence of local order. The $\gamma$ form is effectively in a long range ordered liquid crystalline state. The absence of spherulites in the $\gamma$ form support the interpretation (see Fig. 3b right inset). Liquid crystalline mesophases have also been reported for other precision polymers^17^.

In contrast to the $\gamma$ form, the $\alpha$ and $\beta$ forms show characteristic features which are commonly found for conventional polymeric crystals. Peaks in the WAXD range and conventional spherulitic structures are observed (Fig. 3b left inset). A suitable method to visualize the differences between $\alpha$ and $\beta$ forms is the analysis of the domain sizes within the unit cell. This can be made using form factor analysis. For a lamellar morphology the variation of the peak intensities of the (h00) reflections has to be analyzed (Fig. 3c,d). The thickness of the crystalline and amorphous layers within the unit cell, ${\hbox {l}}_A$ and ${\hbox {l}}_C$, is determined by fitting ${\hbox {I}}_{h00}$ with the function $I = B \times \sin ^2 (q \times l_A) / (q \times l_A)^2$, where B is a weighting factor and ${\hbox {l}}_A$ is the thickness of the amorphous phase. The thickness of the crystalline phase ${\hbox {l}}_C$ is calculated from the difference between spacing d and ${\hbox {l}}_A$. For a 50–50 composition of the two phases even numbered reflections would be suppressed. The form factor analysis of $\alpha$ form of UDAPS16 yields two layers of thicknesses 1.94 nm and 5.41 nm respectively, whereas that of $\beta$ form yields two layers of thicknesses 1.92 nm and 5.08 nm respectively. The 5.41 nm domain of the $\alpha$ form and 5.09 nm domain of form $\beta$ seem to correlate well with twice the length of the UDAPS16 monomer in an all extended conformation. We infer that this domain consists of crystalline monomeric repeating units with the methylene sequences in an all-trans conformation having a length $l^{\alpha }_{C} \approx 2.7 \, \hbox {nm}$ and $l^{\beta }_{C} \approx 2.54 \, \hbox {nm}$ while the smaller domain is formed by primarily amorphous monomeric repeating units. Here, the terms crystalline and amorphous are used in relation to the packing of the methylene sequences because of their relatively longer lengths as compared to the DAP groups. Note that a simplistic calculation based on the layer spacing of the liquid crystalline $\gamma$ form also yields similar results. The assumption that the layered superstructure in the $\gamma$ form ($d^{\gamma } \approx 5.64 \, \hbox {nm}$) is composed of three amorphous monomeric repeating units along the layer, gives a value of $\approx 1.88 \, \hbox {nm}$ per amorphous monomeric domain. This agrees quite well with the value of $l^{\alpha }_{A} \approx 1.94 \, \hbox {nm}$ and $l^{\beta }_{A} \approx 1.92 \, \hbox {nm}$ obtained for the amorphous domains of the $\alpha$ and $\beta$ forms respectively from the form factor considerations. The analysis of the $\alpha$ form of UDAPS18 (refer Supplementary Information) yielded similar values and are listed in Table 1. Based on this discussion, it is evident that the polymeric chain crystallizes in such a way that every third monomer block along the axis is amorphous in the $\alpha$ and $\beta$ forms.

**Table 1:** Structural parameters of UDAPS16 and UDAPS18 at $30^{\,\circ } \hbox {C}$.

	Form	Layer spacing (*d*)	Coherence length ($L_{\textit{coh}}$)	$l_{C}$	$l_{A}$
		(nm)	(nm)	(nm)	(nm)
UDAPS16	$\alpha$	7.35	78.54	2.7	1.94
	$\beta$	7	86.07	2.54	1.92
	$\gamma$	5.64	62.8	–	1.88
UDAPS18	$\alpha$	7.69	88.4	2.86	1.94
	$\gamma$	5.82	67.5	–	1.94

**Figure 4:**
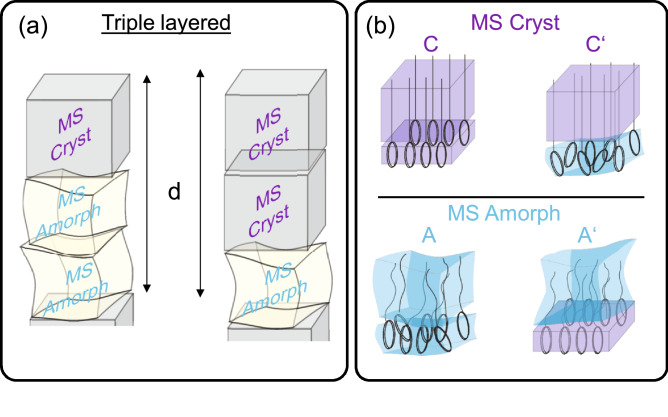
(**a**) Building block model for triple layered layered structures for UDAPS16/18. (**b**) Shows the two modifications of the amorphous and the crystalline blocks depending upon the packing of the individual sub-units within the layers. Note that the classification of the blocks as crystalline or amorphous has been done on the basis of the packing state of the methylene sequences (MS).

Based on these findings, a building block model is used to illustrate this unique behavior and is shown in Fig. 4a. Based on the observed periodicites of UDAPS16 and UDAPS18, it is clear that they exhibit a ’triple layered structure’, where the layered structure contains three monomeric repeating units along the chain axis. Each block consists of a set of monomeric sequences either in the crystalline or the amorphous state. The length of each crystalline block $l_{C}$ and amorphous block $l_{A}$ for the $\alpha$ and $\beta$ forms of UDAPS16 and $\alpha$ form of UDAPS18 estimated from the form factor considerations. Note that the term $l_{C}$ and $l_{A}$ refers to the length of the block along a direction parallel to the polymeric chain axis (or along the surface normal of the layer planes). The nomenclature to classify a block as crystalline or amorphous is based on the state of the methylene sequences which is in line with the conventional approach for classifying precision polyethylenes as crystalline or amorphous.

Although both the $\alpha$ and $\beta$ forms show a triple layered structure with two crystalline blocks followed by one amorphous block, the differences in their periodicities are most likely a product of different packing of the DAP units within the crystalline blocks. A slightly different packing of the rings within the layers, could explain relatively small difference between $l^{\beta }_{C}$ and $l^{\alpha }_{C}$. Here we define two type of crystal blocks, *C* and *C*′. In both cases, the methylene sequences show a similar crystalline packing while a different packing of the rings (e.g. crystalline-C or disordered-C′) might be the reason for the minor difference between $l^{\alpha }_{C}$ and $l^{\beta }_{C}$.

The remarkable agreement in the $l_{A}$ values calculated on the basis of form factors and from the layer spacing of the $\gamma$ form for both UDAPS16 and UDAPS18 provides strong support for the proposed building block model. The interpretation that $\gamma$ is a liquid crystalline-like layered phase comprising of three conventionally amorphous blocks also seems to fit within this framework. A different packing arrangement of the rings in every third block in the $\gamma$-form could explain the observed three-fold periodicity. Two modifications of the amorphous block, *A* and *A*′ are possible based on the packing of the rings as shown in Fig. 4b (e.g. disordered rings for A, crystalline rings for A′). It is also not unexpected that the rings can crystallize but the methylene sequences remain amorphous^20^. The background is that rings commonly tend to crystallize at much higher temperatures as compared to methylene sequences. Once the layers contain ring-like sub-units, crystallization of the methylene sequences is hindered and can be even completely prevented. In thymine end-functionalized polyethylene^34^, the crystallization of the thymine end-groups reportedly prevented the crystallization of the methylene sequences, although preserving a long range ordered lamellar morphology due to the crystallization of the rigid thymine units within planes. Hence, the claim that every third DAP-ring plane could pack in a crystalline or amorphous manner seems quite plausible. Within this framework, the superstructures could be an assembly of a combination of the shown building blocks. The $\alpha$ form is most likely is a sequence of type *CCA* or *CCA’*, the $\beta$ form is of type *C’C’A* or *C’C’A’* and the $\gamma$ form is either *AAA’* or *A’A’A*. Considering the length scales observed it is clear that the proposed triple layered structural model gives a consistent picture and a common explanation for the observed structural features. The indication that the sub-units viz-DAP rings and methylene sequence can pack differently within their layers also points towards a certain competition of the packing tendency of the sub-units. Such competition of the packing tendencies of individual sub-units could be of major importance for complex polymeric systems including those discussed here in order to design new advanced polyethylene-based sequence controlled materials.

## Conclusions

X-ray diffraction experiments on three precision polymers with a 2,6 diaminopyridine group placed after every 16th, 18th and 20th   ${\hbox {CH}}_{2}$ unit of a linear polyethylene chain reveal the occurrence of different layered superstructures incorporating three monomers along the chain oriented parallel to the surface normal of the layers. A building block model is proposed explaining the existence of two polymorphic forms, $\alpha$ and $\beta$, wherein two crystalline layers containing crystalline methylene stems are followed by one with amorphous methylene sequences. This arrangement of the blocks is highly regular based on the estimated coherence lengths. We think that constraints induced by the strongly interacting DAP groups play a significant role for the periodic appearance of amorphous methylene sequences along a chain with precisely defined architecture. A triple-layered liquid crystalline-like $\gamma$ phase was also observed. A rational design of precision polymers where the energetic contributions of methylene sequences and the functional groups compete can be used to optimize the layered superstructures. Methylene sequence length, functional group interactions, thermal treatment are understood as possible strategies to tailor the long range ordered layered morphology. This approach may open new possibilities regarding property optimization in this novel class of polymeric materials.

## Materials and methods

### Polymers

A series of linear precision polymers containing a 2,6-diaminopyridine (DAP) group after every 16th (UDAPS16), 18th (UDAPS18) and 20th (UDAPS20) ${\hbox {CH}}_{2}$ unit was synthesized using ADMET polymerization technique. The detailed synthesis route is described in Refs.^35,36^. The molecular weights of the sample are in the range 5–20 kg/mol as taken from secondary information and related systems^35,37^. Direct information from GPC is inaccessible since no suitable solvent is found for the investigated samples. Significant fractions of very small oligomers ($\le 1 \, \hbox {kg/mol}$) can be definitively excluded based on NMR and MS data.

### X-ray scattering

X-ray scattering experiments on powder samples were performed in transmission mode using a SAXSLAB laboratory setup (Retro-F) equipped with an AXO microfocus X-ray source with an AXO multilayer X-ray optic (ASTIX) as monochromator for ${\hbox {Cu K}}_{\alpha }$ radiation $(\lambda = 0.154 \, \hbox {nm})$. A DECTRIS PILATUS3 R 300K detector was used to record the 2D scattering patterns. As sample holders two millimeter thick aluminum discs with a central hole having a diameter of 1.5 mm were used. A twin slit system was used for the measurements with slits of diameter 0.9 mm and 0.4 mm. The measurements were performed during cooling and subsequent heating using a Linkam hot stage between 30 and $180^{\,\circ } \hbox {C}$ with a temperature step of 10 K.

### Polarized optical microscopy

An Olympus BX51 polarized optical microscope was used to capture the spherulitic growth of the polymers. The sample temperature, heating and cooling rates were controlled using a LINKAM TP94 hot stage. All samples were heated to $170^{\, \circ } \hbox {C}$ prior to crystallization. During isothermal crystallization, a cooling rate of 90 K/min was used to cool the sample from the melt to the crystallization temperature.

## Supplementary information

Supplementary Information
