# Supplementary material for: Tuning layered superstructures in precision polymers
Source: Sci Rep. 2020 Jul 21;10:12119. doi: 10.1038/s41598-020-68927-x (PMC7374706; doi:10.1038/s41598-020-68927-x)
Supplement: Supplementary file 1 — Supplementary Information [file 41598_2020_68927_MOESM1_ESM.pdf]

SUPPLEMENTARY INFORMATION

Tuning layered superstructures in precision polymers

Varun Danke,[†,¶] Sophie Reimann,[‡] Wolfgang H. Binder,[‡] Gaurav Gupta,[*,†,¶] and Mario Beiner[*,†,¶]

Fraunhofer Institute for Microstructure of Materials and Systems IMWS, Walter-Hülse-Straße 1, D-06120 Halle (Saale), Germany, and Institute of Chemistry, Chair of Macromolecular Chemistry, Faculty of Natural Sciences II, Martin-Luther-University Halle-Wittenberg, D-06120 Halle (Saale), Germany

E-mail: gaurav.kumar.gupta@imws.fraunhofer.de; mario.beiner@imws.fraunhofer.de

[*]To whom correspondence should be addressed

[†]Fraunhofer Institute for Microstructure of Materials and Systems IMWS, Walter-Hülse-Straße 1, D-06120 Halle (Saale), Germany

[‡]Institute of Chemistry, Chair of Macromolecular Chemistry, Faculty of Natural Sciences II, Martin-Luther-University Halle-Wittenberg, D-06120 Halle (Saale), Germany

[¶]Faculty of Natural Sciences II, Martin-Luther-University Halle-Wittenberg, Heinrich- Damerow-Straße 4, D-06120 Halle (Saale), Germany

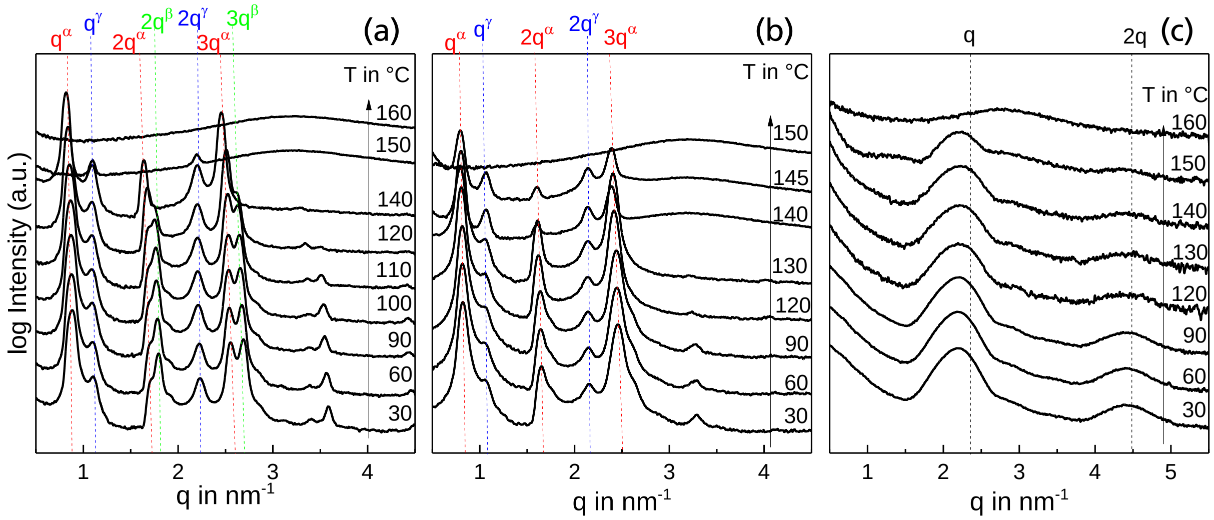

S. 1: Temperature dependent intermediate angle X-ray diffraction patterns (IAXD) during heating in UDAPS16 (a), UDAPS18 (b) and UDAPS20 (c). The curves are vertically shifted for the purpose of visibility.

S. 1 shows the temperature dependent IAXD patterns during heating. As discussed in the main text, during cooling the α form forms first in UDAPS16 and 18 followed by the γ. However during heating, the α form melts at lower a temperature than the γ. This is clearly visible for UDAPS16 (a). In UDAPS18 (b) the intensities of q^α, $2q^\alpha$ and $3q^\alpha$ are substantially reduced in comparison to that of q^γ and $2q^\gamma$ at 145 °C prior to melting. Form α in UDAPS18 has a lower melting temperature than the γ, however, both melt within a 5 °C window. Hence pure γ form is not seen here as seen in the case of UDAPS16 (a). Additionally, the partial β-α solid-solid transition in UDAPS16 is reproducible during heating within the same temperature window 90 °C- 120 °C. UDAPS20 shows just one layered state as recorded during cooling. In UDAPS20, the long period is seen at q≈0.44 nm^{-1} at room temperature corresponding to an interplanar spacing of 12.8 nm. This is quite close to the coherence length 12.3 nm (main text fig 2 (b)).

S. 2 shows representative peak fittings of the third order layer reflections for the α and β forms during cooling (left panel) and subsequent heating (right panel) using a Gaussian fit. The obtained parameters have been used for the evaluation of the layer spacings, coherence lengths and intensities depicted in the main text. The β-α solid-solid transition appears

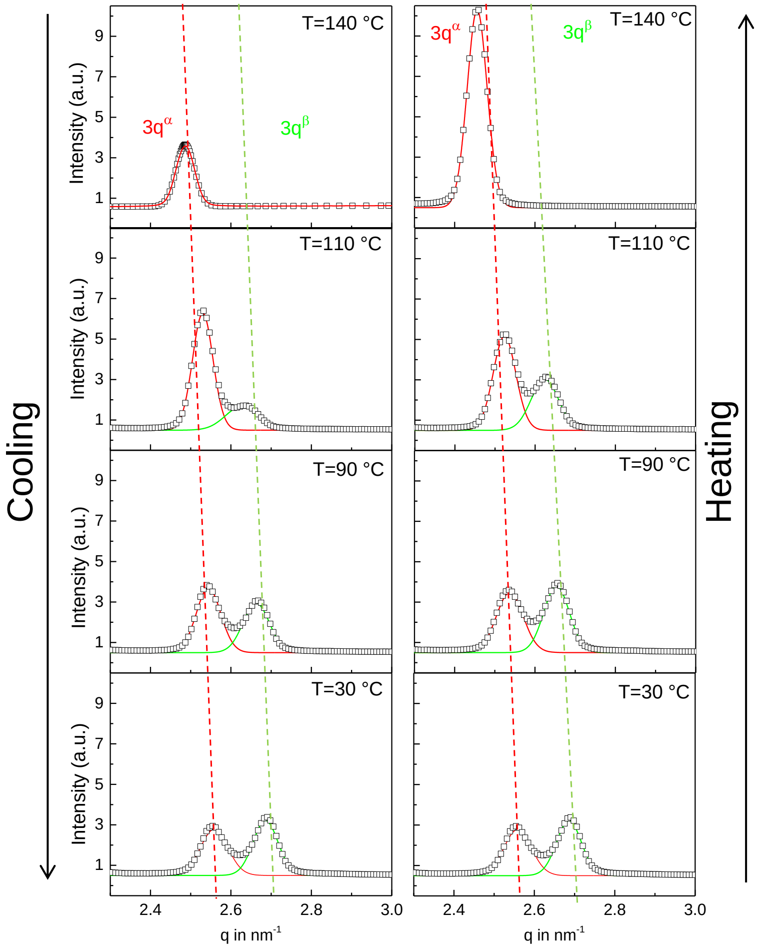

S. 2: The left panel shows the deconvolution of the third order layer reflections $3q^{\alpha}$ and $3q^{\beta}$ using a Gaussian fit at representative temperatures during cooling in order to get the parameters peak width, peak position and integrated intensity. The right panel shows the same during heating. Note that both the x- and y-axes for all the plots are identical.

in the same temperature window during heating as in cooling and is apparent from the disappearance of the $3q^{\beta}$ reflection and a simultaneous increase in the $3q^{\alpha}$ reflection.

S. 3(a) shows IAXD patterns for UDAPS18 at selected temperatures during heating. The pattern at 30 °C shows the presence of both the α and γ forms. At 145 °C, the reduction in the intensities of the q^{α}, $2q^{\alpha}$ and $3q^{\alpha}$ is visible whereas the intensities of q^{γ} and $2q^{\gamma}$ show no visible change hinting that the α form is gradually melting whereas the γ form is fairly stable at this point. The reduction in the peak intensities of the α form in the IAXD patterns are complemented by an almost complete disappearance of WAXD peaks (S. 3(b)). Although it is clear that the α forms melts prior to the γ form as in UDAPS16, a IAXD/WAXD pattern

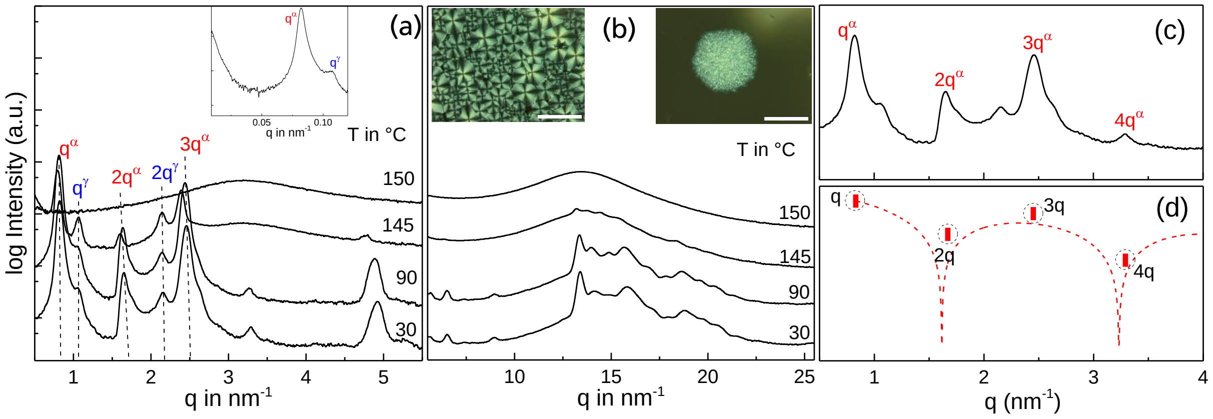

S. 3: (a) IAXD patterns at specific temperatures during heating showing the presence of two layered states in UDAPS18. The inset shows a measurement performed in the lower q range highlighting that no conventional SAXS peak corresponding to the long-period is present. (b) shows corresponding WAXD patterns at the respective temperatures. The left and right insets show polarized optical micrographs for samples isothermally crystallized at 30 °C and 150 °C respectively with a scale bar of 200 μm. (c) shows the IAXD pattern at 30 °C. (d) shows the lamellar form factor fit of the peak intensities for the α form.

for pure γ in UDAPS18 is not seen on account of the small window of 5°C in which both the forms melt. The observations for UDAPS18 also support our claim that the γ form is a liquid crystalline state with a long range layered order but no lateral order between the chains. The differences in the spherulitic structures of the α and γ forms seen in UDAPS16 are also seen for UDAPS18. The coarser structures observed in the γ form indicate a liquid crystalline order (S. 3(b) right inset).

Calculation of amorphous and crystalline blocks for UDAPS18 can be done in a similar way as described in the main text for UDAPS16 (S. 3(c) and (d)). A lamellar form factor fit yields the lengths of the crystalline blocks l_C^α=2.87 nm which corresponds fairly well with the length of the UDAPS18 monomer (2.88 nm) while the length of the amorphous block was found to be l_A^α=1.94 nm for the α form. By using the layer spacing of the γ form it also possible to estimate l_A^α. Dividing the layer spacing of the γ form (5.82 nm) by three, the average length of the amorphous block is found to be l_A^α=1.94 nm which is in excellent agreement with the l_A^α obtained from the form factor fit confirming the triple layers and the building block hypothesis.

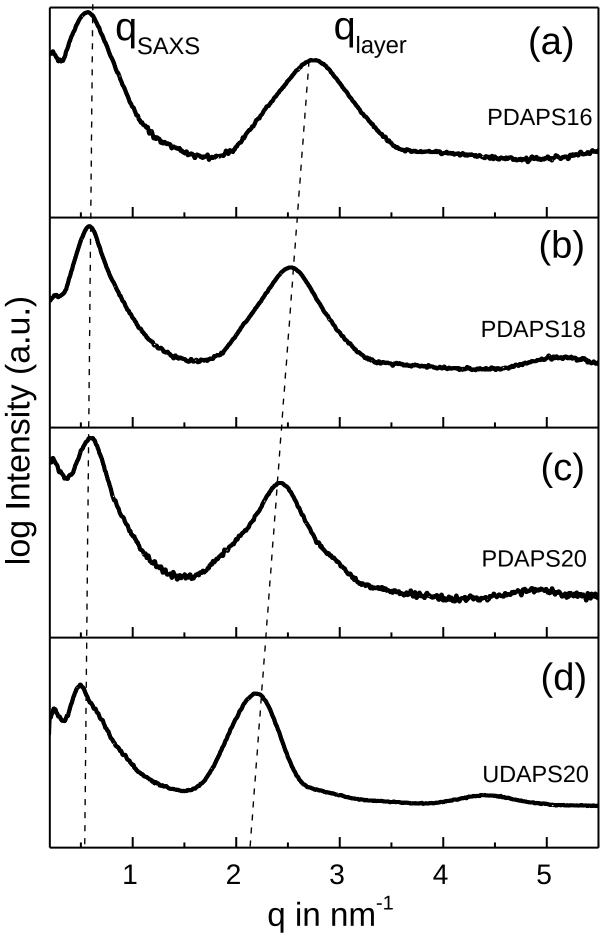

S. 4: IAXD patterns for (a) PDAPS16, (b) PDAPS18(b), (c) PDAPS20 and (d) UDAPS20 are shown to highlight their structural similarities

The IAXD patterns for the polymers containing methyl protected DAP groups also (PDAPS) show a layered morphology (refer S. 4). The replacement of the H-atom by a methyl group prevents the formation of hydrogen bridges between the neighboring DAP units. The calculated layer spacings of 2.29 nm, 2.47 nm and 2.61 nm for PDAPS16, PDAPS18 and PDAPS20 respectively indicate that the layer consists of one monomer along the layer normal (calculated monomer lengths of 2.63 nm, 2.88 nm and 3.13 nm respectively for PDAPS16, PDAPS18 and PDAPS20). The patterns are quite similar to the one observed in UDAPS20. The relatively broad reflections also indicate a substantially reduced structural order in comparison to UDAPS16 and UDAPS18. In addition, a conventional SAXD peak corresponding to the long period is also seen for all the three methyl protected

analogs as well as the UDAPS20 at a similar position indicating the presence of inter-layer amorphous region. These observations support the claim that the triple layered superstructures in UDAPS16 and UDAPS18 are a product of a higher concentration of the strong interactions between the DAP groups. These structural similarities and differences from this direct comparison highlight the importance of the role of the supramolecular interactions in governing the structure formation process.
